# Engineering of Janus-Like Dendrimers with Peptides Derived from Glycoproteins of Herpes Simplex Virus Type 1: Toward a Versatile and Novel Antiviral Platform

**DOI:** 10.3390/ijms22126488

**Published:** 2021-06-17

**Authors:** Annarita Falanga, Valentina Del Genio, Elizabeth A. Kaufman, Carla Zannella, Gianluigi Franci, Marcus Weck, Stefania Galdiero

**Affiliations:** 1 Department of Agricultural Sciences, University of Naples “Federico II”, Via Università 100, Portici, 80055 Naples, Italy; annarita.falanga@unina.it; 2 Department of Pharmacy and CIRPEB, University of Naples “Federico II”, Via Montesano 49, 80131 Naples, Italy; valentina.delgenio@unina.it; 3 Department of Chemistry and Molecular Design Institute, New York University, New York, NY 10003, USA; eak393@nyu.edu (E.A.K.); mw125@nyu.edu (M.W.); 4 Department of Experimental Medicine, Second University of Naples, Via de Crecchio 7, 80138 Naples, Italy; carlazannella88@gmail.com; 5 Department of Medicine, Surgery and Dentistry “Scuola Medica Salernitana”, University of Salerno, 84081 Baronissi, Italy; gfranci@unisa.it

**Keywords:** antiviral compounds, dendrimers, peptides

## Abstract

Novel antiviral nanotherapeutics, which may inactivate the virus and block it from entering host cells, represent an important challenge to face viral global health emergencies around the world. Using a combination of bioorthogonal copper-catalyzed 1,3-dipolar alkyne/azide cycloaddition (CuAAC) and photoinitiated thiol–ene coupling, monofunctional and bifunctional peptidodendrimer conjugates were obtained. The conjugates are biocompatible and demonstrate no toxicity to cells at biologically relevant concentrations. Furthermore, the orthogonal addition of multiple copies of two different antiviral peptides on the surface of a single dendrimer allowed the resulting bioconjugates to inhibit Herpes simplex virus type 1 at both the early and the late stages of the infection process. The presented work builds on further improving this attractive design to obtain a new class of therapeutics.

## 1. Introduction

Outbreaks and pandemic transmission of viruses, such as coronaviruses and influenza viruses, set off a global health emergency across the world. The recent COVID-19 pandemic has demonstrated its catastrophic impact worldwide on human health and on socioeconomic growth [1]. A major challenge against infections triggered by many viruses is the lack of effective methods for prevention and treatment. Nanotechnology provides the foundation for the advancements in antiviral strategies [2]. In fact, several unique features of nanomaterials (such as small-size, high surface-to-volume ratio modifiable surfaces) may contribute to favor multiple antiviral effects, which may include virus inactivation and blocking a virus from entering host cells.

Synthetic polymer scaffolds have played a primary function in the progress of modern biomedicine. Not only have macromolecules exhibited antimicrobial, antifouling, and stimuli-responsive properties [3,4,5], there are prominent examples of biocompatible, yet fully synthetic, systems that function in drug delivery, tissue engineering [6,7], and the fabrication of bone growth scaffolds [8,9]. The development of controlled polymerization routes such as ring-opening polymerization [6,10,11,12], ring-opening metathesis polymerization [13], and controlled radical polymerization methods [14,15,16,17] has allowed for the synthesis of well-defined and multifunctional scaffolds. Multiple polymer systems such as poly(lactide) [18,19,20,21], poly(norbornene) [22,23], and poly(benzyl-L-glutamate) [24,25,26] have been exploited to engineer functional architectures such as polymer–protein conjugates [27,28,29,30,31,32,33], polymer vesicles [34,35,36,37,38], drug delivery scaffolds [26,34,39], and organic nanoparticles [40].

Dendrimers are a discrete and sequence-specific platform capable of securing multiple orthogonally bound functionalities (e.g., dyes, peptides, drugs) within a well-defined and tailored arrangement. Dendrimers, products of an iterative synthesis with discrete monomer building blocks, are functional nano/macromolecules owing to a well-defined size, tailored structure, and precise number of active termini that dictate their properties and applications [41]. While groundbreaking work on dendrimers was accomplished over 30 years ago, dendrimers [42,43,44,45] appear to be one of the most attractive synthetic architectures for many disciplines with the most effective applications ranging from transdermal drug delivery [46], gene delivery [47], and as magnetic resonance imaging contrast agents [48], to dendritic sensors and safe and effective microbicides [49,50]. Dendrimers represent one of the most promising drug delivery scaffolds (DDS) [51] for targeted drug delivery.

Dendrimers are monodisperse with a perfectly branched architecture [52,53]. As they grow in generation, the number of termini exponentially increases, while only linearly increasing in radius. Thus, the termini become more densely packed, giving the entire structure a globular shape wherein the termini radiate outwards from a central core. Monodispersity and globularity along with multivalency, self-assembling capacity, electrostatic interactions, chemical stability, low cytotoxicity, and aqueous solubility are all features that continually reveal dendrimers to be a prominent choice in the biomedical field [54]. Dendrimers appear to be also an appropriate carrier for the delivery of antiviral therapeutics thanks to their controllable release potency and improved translocation across epithelial and endothelial barriers; moreover, the presence of multiple peripheral functional groups facilitates a higher binding affinity towards surface viral proteins.

Herein, we report the synthesis of two functional Newkome-type dendrimer scaffolds, comprised of monotelechelic and heterotelechelic termini that are engineered for post-synthetic bioconjugation of selected peptides. We exploit the valency of the resulting monofunctional and Janus-type architectures to anchor antiviral peptide sequences derived from the envelope fusion-glycoproteins (gH and gB) of Herpes simplex virus type 1 (HSV-1). HSV represents one of the major global health problems; over the past 40 years, numerous strategies have been developed to fight the herpetic infection and three classes of drugs are licensed for HSV treatment, all based on the inhibition of viral DNA replication such as acyclovir (ACV), cidofovir, and foscarnet [55]. Although these agents are efficacious against the HSV infection, side effects and limitations are associated with their use. Importantly, the resistance, especially among immunocompromised patients undergoing long-term therapy, represents an important clinical problem [56]. Therefore, it is of utmost importance to identify alternative antiviral systems with different mechanisms of action and different targets [57].

The emergence of virus-derived peptides as new antiviral agents has strongly impacted anti-HSV research. Peptides have several advantages; indeed, they can be both highly specific and effective, while their inherent biodegradability limits their overall accumulation in tissues, resulting in lower toxicity. Although several drawbacks are associated to their use such as short half-life, potential immunogenicity [58], and high production costs, the design of peptidomimetics can overcome these issues. This work proposes the combination of dendrimer and peptide chemistry into the formation of a new biomedical scaffold able to combat HSV with different mechanisms during the infection process.

HSV-1 enters cells through fusion of the viral envelope with the plasmatic membrane of the host cell in a flow of molecular interactions involving multiple viral glycoproteins and cellular receptors [59]. The envelope glycoproteins gH/gL, gB, and gD are all essential for the entry process, and the four glycoproteins are able to induce the fusion of cellular membranes in the absence of virus infection [60]. The present paradigm of entry foresees gD-receptor binding/signalling gH/gL to trigger gB to mediate fusion. The trimeric fusion protein gB inserts into the host cell membrane and refolds to fuse the viral and cellular membranes, allowing the viral capsid and genome to enter the host cell [61]. Prior to receptor binding, the C-terminus of the gD ectodomain occludes the receptor-binding site. gD receptor binding displaces the gD C-terminus and transmits a signal to activate gH/gL, which then transmit this signal to trigger the fusion protein gB. Eventually, gB inserts its fusion loops into the host cell membrane and collapses back on itself, thereby fusing the viral and cell membranes [61,62]. Likely, more than one gB trimer must be triggered to create a fusion pore, which allows the viral capsid to enter the cell.

Peptides interfering with any of these steps influence viral penetration and may present a considerable inhibitory activity with low toxicity. As a matter of fact, we previously reported the antiviral activity of several peptides derived from glycoproteins gH and gB, which may interfere with the many conformational changes of these glycoproteins, ending in viral penetration [63,64,65,66,67]. We analysed the antiviral activities of the peptide gH493-511 and its longer version gH493–537 [65]. Results showed a higher activity of the shorter sequence (IC_50_ = 160 mM) and an ability to inhibit infectivity when present during virus attachment-entry into cells; in fact, no activity was observed when cells were infected with HSV-1 for 45 min and only afterwards the peptide was added to the inoculum and in cell-preexposure experiments. A scrambled version did not inhibit HSV-1 infectivity under analogous experimental conditions and the peptide was shown to be specific as no activity was revealed with unrelated enveloped viruses such as parainfluenza-2 virus [65]. Furthermore, with the purpose of interfering with conformational changes, we previously developed a set of peptides designed on the amino acid sequence of the long helical region present in the post-fusogen structure of gB and we developed some peptide sequences that were highly effective in inhibiting the virus thanks to their ability to trap gB in its pre-fusogenic state and impede the refolding process from pre- to post-fusion state [62,67]. These data make gB peptide analogues attractive candidates for further drug development against HSV-1 and, analogously to other viruses, we also demonstrated that membrane targeting through cholesterol conjugation significantly enhanced the antiviral potency of our prototype inhibitors [68].

Nonetheless, antiviral peptide activity relies on a reversible binding event, which may reduce their usefulness in vivo. In fact, in vivo dilution causes a loss of binding and the release of an unaltered virus particle, which is again infective. Recently, it was reported that strong multivalent binding [69] may head to local distortion and consequent virus deformation, which cause irreversible loss of infectivity. We explored the likelihood to produce an irreversible deformation of viral particles through the multivalency of dendrimers and the use of two different peptide sequences (one derived from gH and the other from gB) with different targeting and inhibition mechanisms.

## 2. Results and Discussion

### 2.1. Synthesis of DendrimerA and DendrimerB

**Figure 1:**
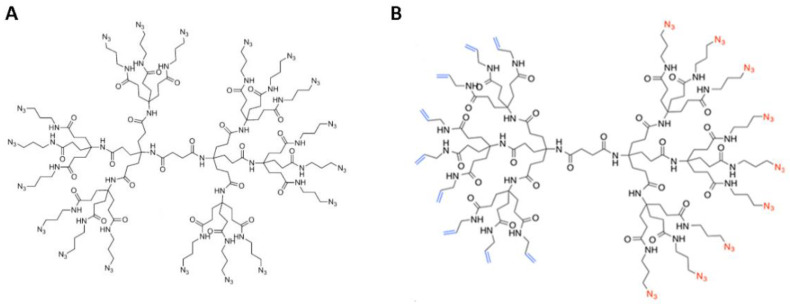
DendrimerA (panel **A**) and Janus-type DendrimerB (panel **B**) used in the study.

Our dendrimer design is based on the Newkome-style dendrimers that are polyamide-based, thus mimicking proteins, assuring biocompatibility, and promoting biodegradability. We have previously reported the syntheses of both the monofunctional dendrimer scaffold (DendrimerA) and the Janus-type dendrimer (DendrimerB) (Figure 1) [70,71] (see also SI with synthetic and purification details of the Janus dendrimer).

### 2.2. Peptide Functionalization of Monofunctional (DendrimerA) and Janus (DendrimerB) Dendrimers

The two peptides (see Table 1 for sequences) were bound to the dendrimer differently. The gB peptide was coupled from the C-terminus. A preliminary antiviral analysis performed on the gB peptide linked to the dendrimer from the N-terminus and the C-terminus confirmed previous results showing that the peptide loses activity when bound from the N-terminus (data not shown). A similar result was also reported by Lombardi et al. [68], indicating that cholesterol tagging was more effective when the C-terminus of the peptide was modified. As for the gH peptide, we evidenced an opposite result with slightly better results when functionalizing the N-terminus (data not shown). For this reason, we decided to bind the gB peptide from the C-terminus and the gH peptide from the N-terminus.

The peptides were synthesized with a propargyl glycine residue (PrA) at the correct terminus to provide a handle for the copper-catalyzed azide/alkyne cycloaddition reaction (CuAAC) with the terminal azides of DendrimerA. The click functionalization was performed in a water/methanol solution (1:1 *v*/*v*) with 2:4 equivalents of CuSO_4_·5 H_2_O:sodium ascorbate. The conjugates were extensively purified through dialysis, HPLC, and ultrafiltration on 30 KDa filters.

**Figure 2:**
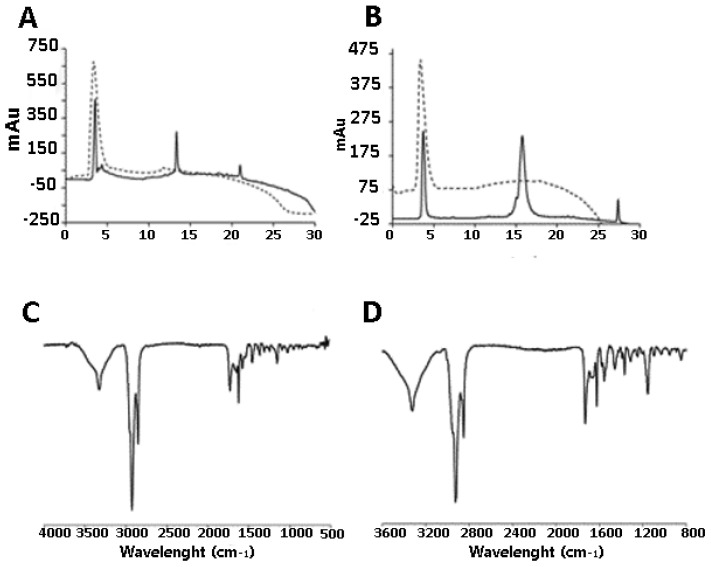
HPLC traces of crude (solid line) and purified (dashed line) peptidodendrimer conjugates, DendrimerA-gH (panel **A**), DendrimerA-gB (panel **B**). IR spectroscopic analysis of DendrimerA-gH (panel **C**) and DendrimerA-gB (panel **D**).

Successful peptide conjugation was confirmed using HPLC through the appearance of new peaks at retention times (RT) different from the controls that were run at the same conditions (Figure 2, panels A and B). The peptide coupling was also confirmed by IR spectroscopic analysis demonstrating the disappearance of the azide stretch at 2098 cm^−1^ suggesting that, within the instrumental error range, azides were consumed and converted in triazoles (Figure 2, panels C and D).

DendrimerB was functionalized with Cys-gH via photoinduced thiol–ene reaction and with gB-Pra via copper-catalyzed azide/alkyne cycloaddition. The thiol–ene reaction was performed with the Cys-gH peptide in DMF-H_2_O in presence of DMPA at 4 °C for 1 h with UV light irradiation. Subsequently, DendrimerB/gH was functionalized with gB as reported above. The conjugates were purified through dialysis and analysed by IR spectroscopy and ζ-potential measurements. IR analysis demonstrated the disappearance of the azide stretch, suggesting that gB was conjugated to DendrimerB and the reduction of the stretching C=C at 1633 cm^−1^ indicating the formation of the bond between Cys-gH and DendrimerB. Furthermore, analysis of the zeta-potential results show that a negative charge was obtained for DendrimerB (−11 mV), while a positive charge was obtained for DendrimerB + gB/gH (+5.17 mV) (Table 2). The net ζ-potential value, observed at pH 7, further supports peptide conjugation on the dendrimer surface.

### 2.3. Circular Dichroism

**Figure 3:**
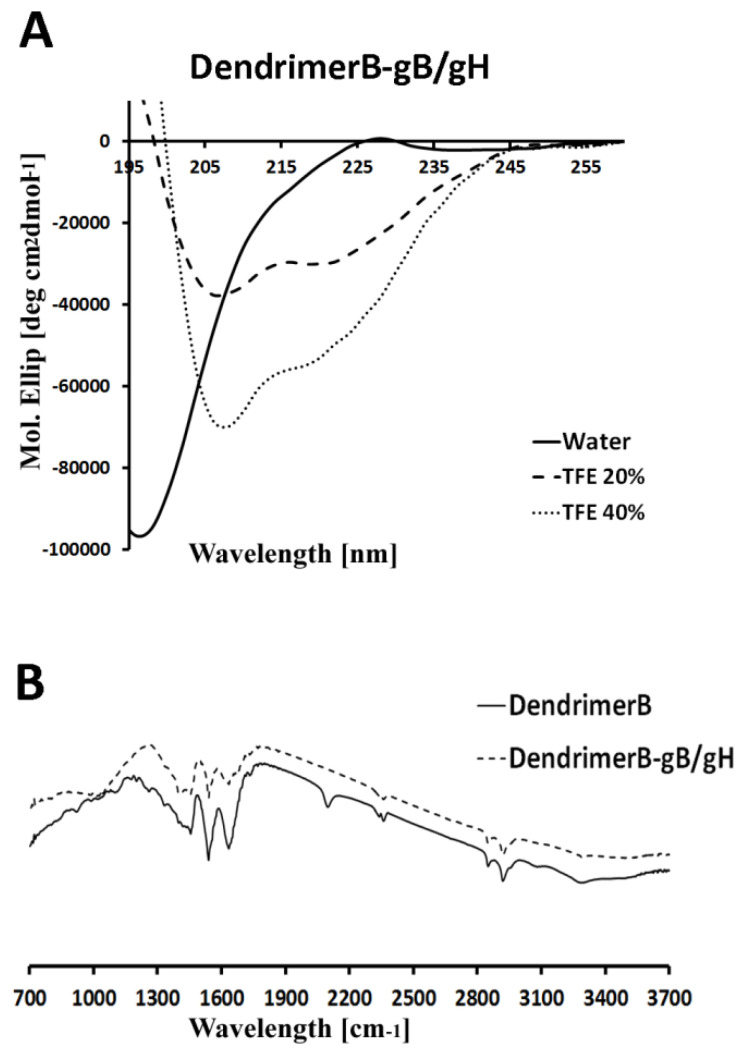
Circular dichroism of DendrimerB + gB/gH in water with different percentage of TFE (panel **A**) and IR spectroscopic analysis of DendrimerB and DendrimerB + gB/gH (panel **B**).

The molecular conformation of DendrimerB + gB/gH was investigated by far-UV CD spectroscopy, which is an excellent technique for rapid determination of the secondary structure (Figure 3A). As expected, the CD spectrum in water indicated a random coil conformation for DendrimerB + gB/gH (Figure 3A). Both peptides gH and gB are random coil in aqueous solution but form an α helix in membrane-mimetic environments [62,65,72]. CD spectra were obtained in several percentages of trifluoroethanol (TFE), which is widely used to simulate the membrane environment. The spectrum of DendrimerB + gB/gH show that the peptides adopt an α helix with minima at approximately 208 and 222 nm. (Figure 3A). The obtained spectra suggest that the secondary structure of the peptides was not disturbed by attachment to a dendrimer.

### 2.4. Cytotoxicity Studies

**Figure 4:**
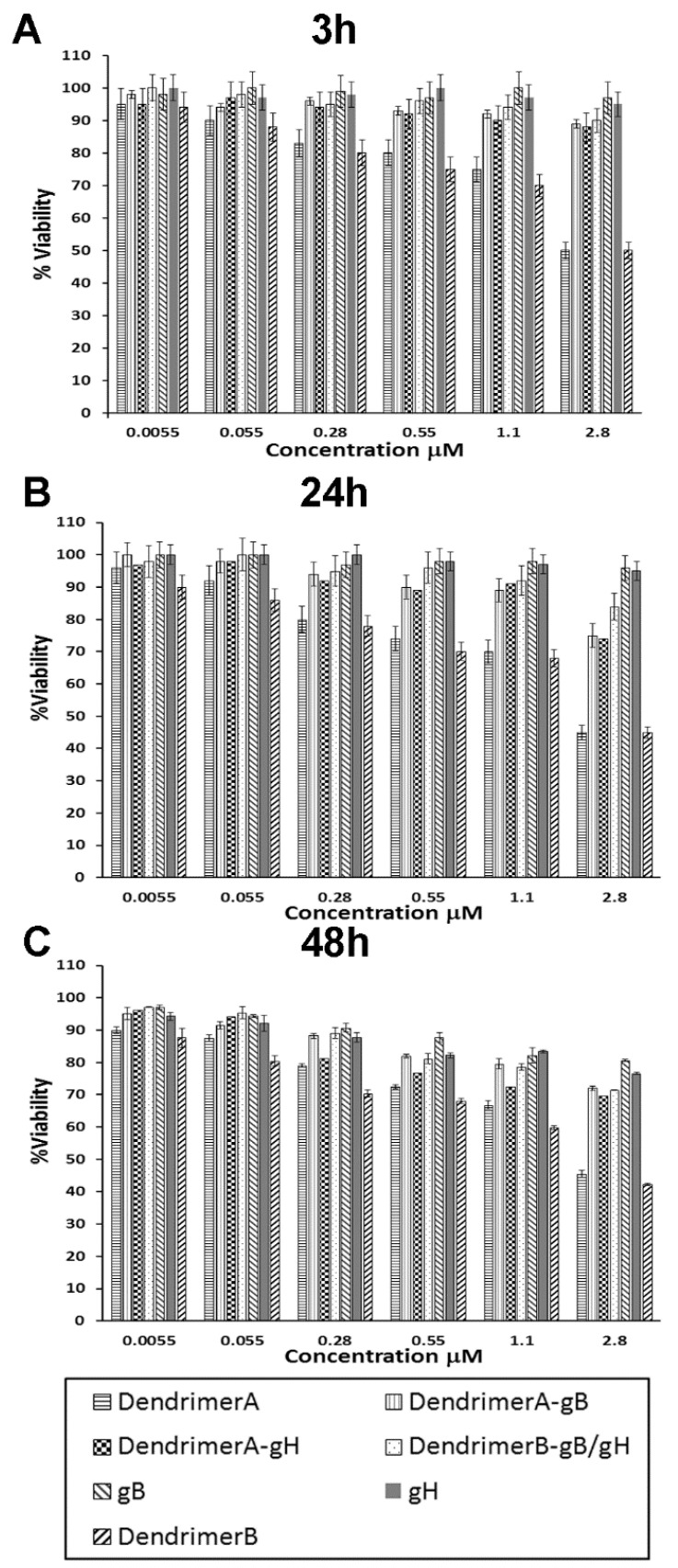
Cytotoxicity experiments performed after 3 h (panel **A**), after 24 h (panel **B**) and after 48 h (panel **C**). Experiments were performed in triplicate, and the percentages of viability were calculated with respect to no-compound control experiments. Error bars represent standard deviations.

To confirm that synthetized peptide dendrimers do not cause toxic effects on cells, monolayers of Vero cells were exposed to different concentrations (5.5 nM, 55 nM, 0.28 μM, 0.55 μM, 1.1 μM, 2.8 μM) of each compound for 3, 24 and 48 h, and cell viability was determined by the MTT assay. No statistical difference was detected between the viability of control (untreated) cells and that of cells exposed to the peptide dendrimers (Figure 4) up to the concentration used in antiviral testing at 3 and 24 h. A small decrease in viability was observed at 48 h. Marginal toxicity was obtained for the dendrimer without the peptides linked to its termini, at concentrations that were considerably higher than those required for antiviral activity.

### 2.5. Antiviral Assays

**Figure 5:**
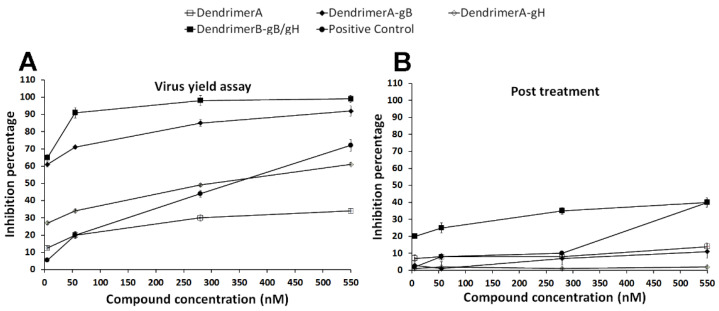
(**A**) Compounds were present in the cell culture during and after viral adsorption (virus yield assay); (**B**) cells were exposed to compounds after virus penetration (post treatment). The control used in both experiments is acyclovir. Experiments were performed in triplicate, and the percentages of inhibition were calculated with respect to no-compound control experiments. Error bars represent standard deviations.

To test whether the peptide dendrimers are able to inhibit HSV-1 in vitro, several experiments were performed. A virus yield reduction assay in which the peptide dendrimers of interest were present in the cell culture during and after viral adsorption was initially performed. The degree of HSV-1 replication was determined by titration of harvested viruses, and showed a consistent decrease in replication efficiency with more than 60% inhibition at a peptide-dendrimer concentration of 5.5 nM for both DendrimerA-gB and DendrimerB + gB/gH, while we observed the same percentage of inhibition for DendrimerA-gH only at 550 nM. Inhibition of HSV-1 replication with the Janus bifunctionalized dendrimer was able to reach 90% already at 55 nM. DendrimerA, without any peptide conjugation, was able to produce an inhibition close to 30% at the highest concentration used (550 nM), suggesting that the dendrimer structure itself grants a certain level of antiviral activity, which is strongly enhanced by the specific peptide sequence coupled to its termini (Figure 5, panel A).

In order to identify the step in the entry process that was being inhibited by our compounds, and thus to understand the mechanism of inhibition, the compounds were tested under different conditions. Our hypothesis was that the Janus dendrimer was able to interfere during the early penetration phase. To exclude the hypothesis of an action inside the cell at a post-entry event, a post-treatment assay was executed by adding the compounds at different concentrations (Figure 5, panel B). No concentrations used in this experiment were able to significantly reduce HSV-1 replication, indicating that both the dendrimer and the peptide dendrimers were ineffective once the viruses had already entered inside the cell. The results described soundly suggest that our compounds target an early step of the HSV infection cycle. Some low activity was observed for the Janus dendrimer (DendrimerB + gB/gH), indicating that the bifunctionalized dendrimer may have access in the cell interior, and thus, also exert its function inside the cell.

**Figure 6:**
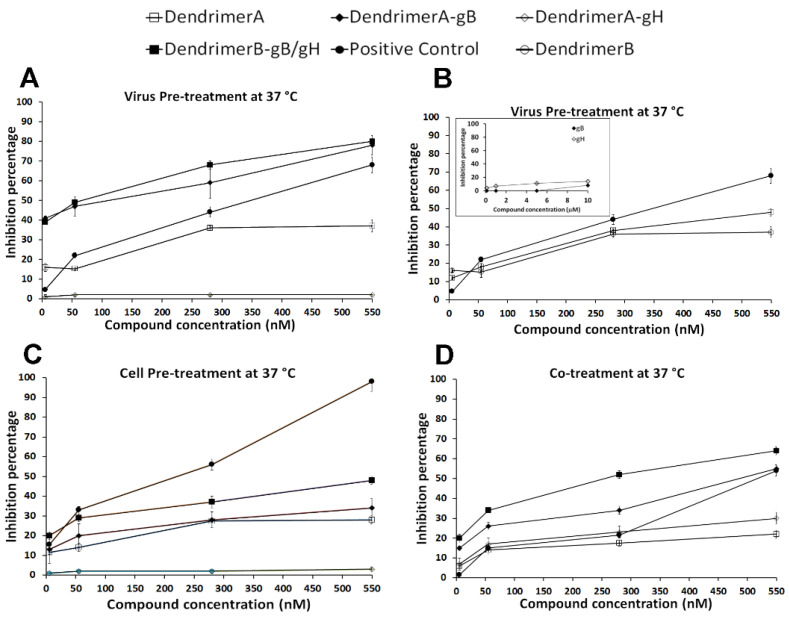
The virus was pre-incubated with compounds for 1 h at 37 °C prior to the addition to the cells (Virus Pre-Treatment, panels **A** and **B**). Cells were exposed to compounds either prior to infection (Cell Pre-treatment, panel **C**) or during attachment and entry (Co-treatment, panel **D**). The control is melittin in virus pre-treatment and co-treatment, and is dextran sulphate in cell pretreatment. Experiments were performed in triplicate, and the percentages of inhibition were calculated with respect to no-compound control experiments. Error bars represent standard deviations.

In addition, other experiments were performed to better clarify the mechanism of infection. As shown in Figure 6, panel A, a significant inhibitory effect was observed when the virus was incubated with compounds and subsequently added to the cells. The highest inhibition was again achieved for both DendrimerA-gB and DendrimerB + gB/gH, while we observed a lower percentage of inhibition for DendrimerA-gH at 550 nM. In panel B, we compared the activities in the same experiment of the two dendrimers: DendrimerA and DendrimerB without peptides. We observed a similar activity for both of them. The insert of panel B shows the activities of the isolated peptides at the concentration present on the dendrimers. The data confirm that the peptides alone are not active at the concentration used in the experiments. Thus, the results of the virus pre-treatment experiment clearly indicate that the functionalization of the dendrimers with peptides gB and gH is able to induce a significant enhancement of activity.

The possibility to interfere with an early penetration step was further explored. Vero cells were pretreated with the target compounds for 30 min at 37 °C before infection. Much lower reduction of infectivity was observed (Figure 6, panel C). In contrast, the dendrimer is retaining its activity. Since the toxicity of the dendrimer is minimal, it is assumed that it may exert an antiviral activity by blocking the cell surface. The lower activity of the peptidodendrimers in this experiment is likely due to the fact that both peptides are involved in the interaction with the virus, as demonstrated by the high inhibition activity showed in the virus pre-treatment experiment.

Since inhibition of HSV penetration is likely the result of a combination obtained by the concerted action of the dendrimer on the cell surface and of the peptides responsible for an interaction with the viral glycoproteins, we performed a co-treatment experiment (Figure 6, panel D). The results obtained from the co-treatment experiment support the key role played by the peptides in regulating the activity of the peptide dendrimer.

The analysis of the obtained results supports the view that, in DendrimerB + gB/gH, the surface of the dendrimer is covered by the two peptides, thus the activity of the dendrimer may be shielded during the inhibition mechanism in favor of the peptide activities.

## 3. Discussion

HSV is responsible for many severe diseases and represents a significant challenge to public health due to the rising problem of drug resistance, which is related to overuse of current drugs, as well as to the deficiency in new drug development strategies by the pharmaceutical industry. Hence, the advance in antiviral drug design against HSV infections represents a step forward the global fight against these viruses. Current strategies involving the use of peptides or dendrimers to block viral entry are characterized by the fact that the inhibition concentration is in the micromolar range and likely the interaction is reversible and sensitive to dilutions, which may reduce their usefulness in vivo. In this contribution, we show that it is possible to enhance the mechanism of inhibition by engineering novel antiviral nanotherapeutics which lead to multivalent binding and interactions with the consequent production of irreversible local distortion and loss of infectivity. In particular, we demonstrate that the use of different peptides (i.e., one derived from gH and the other from gB) with different targeting and inhibition mechanisms on multifunctional dendrimers likely determines a deformation of viral particles at much lower concentrations. The existence of several targets on the viral and/or the cell surfaces supports the multivalent binding inhibition strategy. Moreover, dendrimers alone inhibit viral penetration; however, the small size of the molecule, and thus the rigidity of the functional groups, reasonably led to the binding of only a few of the target groups, resulting once again in weak and reversible interactions. In our nanotherapeutics, the peptides perform a key role and, being the surface of the dendrimer covered by them, the activity of the dendrimer is likely shielded during the inhibition mechanism in favor of the peptide activities. Furthermore, the concentration range of activities of the peptides can be significantly reduced when combining them on a dendrimer structure. The idea of selecting different peptides with dissimilar mechanisms of inhibition may also enhance the activity of the nano-compound; the multiple binding to viral particles is useful in trapping and inactivating the virus. We believe that the approach presented here represents a first step towards the development of a novel strategy which has a chance to produce medically relevant drugs to fight many worldwide threatening viral infections. Additionally, in-depth in vitro *and* in vivo experimentations are necessary to establish whether outstanding inhibitory activity over HSV-1 is confirmed. In any case, it should be highlighted that the approach proposed is fundamentally broad spectrum, supporting the potential prevention and treatment of multiple viral infections simply changing the peptides coupled to the dendrimer, which represents a great advantage when unexpected infections occur.

## 4. Materials and Methods

### 4.1. Materials

All chemicals were purchased from Sigma-Aldrich, Acros Organics, Alfa Aesar, or TCI international, and used as received, unless otherwise noted. Dendrons 1 and 6 were synthesized as previously reported from starting materials purchased from Frontier Scientific. 3-Azidopropylamine was synthesized as previously reported. Dialysis membranes (SpectraPor 6) were purchased from Spectrum Labs and used after rinsing the membrane in water for 30 min. LCMS data were recorded on an Agilent LCMSD Trap XCT spectrometer using electrospray ionization (ESI) and methanol as eluent for starting compounds and acetonitrile (0.1% TFA) for species purified by HPLC. ^1^HNMR spectra were recorded using a Bruker AV-400, -500, or -600 spectrometer (400.1 MHz, 500.2 MHz, or 600.2 MHz); ^13^CNMR spectra were recorded on a Bruker AV-600 spectrometer (150.9 MHz). Deuterated solvents were purchased from Cambridge Isotope Laboratories. High-performance liquid chromatography (HPLC) purifications were run on a LC-8A Shimadzu (Kyoto, Japan) system equipped with a SPD-10Avp UV/Vis detector with reverse-phase Jupiter 10u C4 300A column (10 × 250 mm) in varying percentages of water and acetonitrile with 0.1% TFA at a rate of 5 mL/min.

### 4.2. Synthesis of Monofunctional Dendrimer

Synthesis of the monofunctional dendrimer scaffold has been reported previously [70]. Briefly, the starting dendron 1 was functionalized at the amino termini with succinic anhydride to achieve the hemisuccinate dendron 2. The two dendrons are convergently coupled using HATU and Hüngin’s base to afford the symmetrical dendrimer 3. After deprotection of the tert-butyl esters, the carboxylic acid groups of dendrimer 4 were subsequently coupled with azidopropylamine to obtain the final monofunctional dendrimer 5, called DendrimerA.

### 4.3. Synthesis of the Bifunctional Dendrimer (Janus)

The synthesis of the Janus-type dendrimer has been reported previously [71]. Briefly, the amino ester dendron 6 was protected at the amine terminus with a 9-fluorenylmethylcarbamate (Fmoc), after which the ester termini were deprotected. Coupling of 3-azidopropylamine and subsequent deprotection of the Fmoc afforded the aminononaazide dendron 9. This was coupled to the hemisuccinate dendron 2 using HATU in the presence of Hüngin’s base and deprotected using formic acid to yield the Janus bifunctional dendrimer 11. Dendrimer 11 was purified using preparative HPLC using a water/acetonitrile gradient. The eluted product was monomodal, indicative of its monodispersity. ^1^H NMR spectroscopic analysis revealed full functionalization, as evidenced by the relative integration of the core protons to the azidopropyl methylene units. The final coupling of dendrimer 11 with allylamine in the presence of HATU and Hüngin’s base afforded the target bifunctional dendrimer 12 with functionalized dendrimer faces (DendrimerB). DendrimerB was purified using dialysis (1000 MWCO) against methanol, and characterized using ^1^H and ^13^C NMR spectroscopy, as well as MALDI-TOF spectroscopy (see SI) [71].

### 4.4. Synthesis of Peptides

Peptide sequences (Table 1) were synthesized on Rink-amide MBHA resin (0.51 mmol/g substitution). Syntheses were performed on a 20 μmol scale. Fmoc-protected amino acids were coupled using the benzotriazol-1-yl-oxytris(pyrrolidino)phosphonium hexafluorohosphate (PyBOP), hydroxybenzotriazole (HOBt), and diisopropylethylamine (DIPEA) method: 4 eq. amino acid, 4 eq. PyBOP, 4 eq. HOBt, and 8 eq. DIPEA relative to resin loading. The coupling steps were run twice for 20 min each. The Fmoc group was deprotected with 30% piperidine in DMF (*v*/*v*). Propargyl glycine residue (PrA) was added at the terminus to provide a handle for the copper-catalyzed azide/alkyne cycloaddition reaction (CuAAC) with the terminal azides of the monofunctional dendrimer, and, when necessary, cysteine residue was added at the peptide terminus to provide thiol–ene reaction with alchene groups of Janus bifunctional dendrimer (DendrimerB). Fmoc-PrA-OH was coupled once for 45 min using 2 equivalents each of PyBOP, HOBt, and 4 equivalents of DIPEA.

Fully synthesized peptides were deprotected from the resin with trifluoroacetic acid (TFA) containing 3.8% (*v*/*v*) water, 2.2% (*v*/*v*) anisole, 5.5% (*v*/*v*) thioanisole and ethandithiol (EDT) 3.5% (*v*/*v*) at room temperature and precipitated into ice cold ether. The precipitate was dissolved in water and lyophilized to obtain the crude peptides. Peptides were purified by reverse-phase HPLC with water (0.1% TFA) and acetonitrile (0.1% TFA) (from 20 to 80% over a 20 min flow of 20 mL/min), as well as checked to exhibit the expected molecular ion on analysis by high-resolution mass spectrometry (HRMS). Pure peptides (higher than 98%) were achieved in good yields (40% for gB peptide and 50% for gH).

### 4.5. Functionalization of Monofunctional Dendrimer

The dendrimer (1 equivalent) functionalization with PrA-peptide (gH and gB) (36 equivalents of each peptide) was performed in a water/methanol solution (1:1 *v*/*v*, about 1 mL) by using 2:4 equivalents (to the azide moiety) of CuSO_4_·5 H_2_O: sodium ascorbate. The reactions were left stirring for 1 h at 40 °C and for 2 days at room temperature. The resulting functionalized dendrimers were dialyzed against water/EDTA with 1000 MWCO membranes overnight, followed by purification by reverse-phase HPLC using a C4 column with water (0.1% TFA) and acetonitrile (Acn) (0.1% TFA) with a flow rate of 5 mL/min. Solvent gradients of 30 to 95% Acn over 20 min for Dendrimer-gH493-511 (DendrimerA-gH) and from 5 to 90% Acn over 20 min for Dendrimer-gB503-523 (DendrimerA-gB) were used.

After HPLC purification, the peptidodendrimers were passed three times through a 30 KDa (MWCO) ultrafiltration membrane using water:MeOH:DMSO 50/45/5. The functionalization yields were confirmed by UV analysis (εgH = 1189 m^−1^ cm^−1^ at λ = 280 nm); (εgB = 1090 m^−1^ cm^−1^ at λ = 280 nm) and compared to the ratio of peptide initially used for the reaction (36 mol peptide per mol dendrimer). From the UV analysis, the peptide functionalization yields were 50% of the equivalents added.

### 4.6. Functionalization of DendrimerB

The Janus bifunctional dendrimer was functionalized with gB via copper-catalyzed azide/alkyne cycloaddition and with Cys-gH via photoinduced thiol–ene reaction. In particular, dendrimer (1 eq), Cys-gH (1.5 eq) and DMPA (0.2 eq) in a 4:1 mixture of DMF-H_2_O were irradiated with UV light (Spectroline model ENF-240C/FE) while stirring for one hour at 4 °C. After this reaction, gB was coupled to the dendrimer via copper-catalyzed azide/alkyne cycloaddition reaction using the same condition as described above. The Janus bifunctional dendrimer was purified using dialysis (1000 MWCO) against water and characterized using IR spectroscopy.

### 4.7. IR Spectroscopy

The samples were analyzed by FT-IR spectroscopy. The FT-IR spectra were recorded on a UV-Vis spectrophotometer (Jasco, Easton, MD, USA). The characteristic peaks of IR transmission spectra were recorded at a resolution of 4 cm^−1^ over a wavenumber region of 400–4000 cm^−1^.

### 4.8. ζ-Potential Measurement

The ζ-potential of DendrimerB and DendrimerB-gB/gH solutions were measured using Zetasizer Nano-ZS (Malvern Instruments, Worcestershire, UK). All measurements were performed at 25 °C in water, at pH 7 in triplicate.

### 4.9. Circular Dichroism

CD spectra were recorded from 195 nm to 260 in a Jasco J-810 spectropolarimeter using a 0.1 cm quartz cell at room temperature under a constant flow of nitrogen gas. Other experimental settings were: scan speed of 5 nm/min, sensitivity of 50 mdeg, time constant of 16 s, bandwidth of 1 nm. Each spectrum was obtained through averaging three scans; spectra were recorded and corrected for the blank. DendrimerB-gB/gH spectra were recorded in water and in presence of 20% and 40%TFE at a concentration of 9 10^−5^ M.

### 4.10. Cells and Viruses

African green monkey kidney cells (Vero) (ATCC CCL-81) were grown in Dulbecco’s Modified Eagle Medium (DMEM) supplemented with 10% fetal bovine serum (FBS), 2 mM L-glutamine and 100 IU/mL of penicillin–streptomycin in a humidified atmosphere with 5% CO_2_ at 37 °C. HSV-1 (strain SC16) carrying a lacZ gene driven by the CMV IE-1 promoter to express β-galactosidase was propagated on Vero cell monolayers.

### 4.11. Antiviral Assays

Antiviral experiments were executed at different concentrations for all compounds (0, 5.5, 55, 280, and 550 nM); the concentrations refer to the molecule, thus the peptide concentration corresponds to the quantity present on each dendrimer molecule, i.e., 18 times the concentrations of the monofunctional dendrimer and 9 times for each peptide for the Janus dendrimer. All experiments were done in triplicate. The infectivity inhibition percentage was calculated by fixing as 0% inhibition, the number of plaques obtained in negative controls (only virus).

To determine the effect of functionalized dendrimers on inhibition of HSV infectivity, cell monolayers were treated in different ways:

1. *Virus yield reduction assay*. Confluent Vero cell monolayers (12-well plates) were washed with phosphate-buffered saline (PBS) and infected with HSV-1 at multiplicity of infection (MOI) of 1 for 1 h at 37 °C. Then, virus inocula were mixed with the antiviral compounds at the concentrations indicated above. Infected cells were washed with PBS, covered with fresh culture medium, and incubated for 48 h; then, they were scraped into culture medium and disrupted by sonication.
2. *Post-treatment assay.* 5 × 10^5^ Vero cells (12-well plates) were incubated firstly with virus (MOI 0.01) for 45 min at 37 °C and then the compounds were added to the cells followed by an additional incubation period of 30 min at 37 °C.
3. *Co-treatment.* In co-exposure experiment, 5 × 10^5^ cells were incubated with peptides and with the viral inoculum at MOI of 0.01 for 45 min at 37 °C.
4. *Cell pretreatment.* In cell pre-exposure experiment, 5 × 10^5^ Vero cells were incubated with compounds for 30 min at 37 °C and subsequently infected with HSV-1 at MOI of 0.01 for 45 min at 37 °C.
5. *Virus pre-treatment.* In virus pre-exposure assay, HSV-1 at MOI of 0.1 was incubated with compounds for 45 min at 37 °C, and then the mixture was titrated on Vero cell monolayers.

For all treatments, non-penetrated viruses were inactivated by citrate buffer at pH 3.0 after 45 min incubation with cells at 37 °C. The cells were then incubated for 24 h at 37 °C in DMEM supplemented with carboxymethyl cellulose (CMC) 5%. The total virus yield in each well was titrated by plaque assay. Plaques were stained with X-gal and microscopically counted. The mean plaque counts for each concentration were reported as a percentage of the mean plaque count compared to the control virus. The number of plaques was plotted as a function of concentration; concentrations producing 50% reductions in plaque formation were determined as the IC_50_.

### 4.12. Cytotoxicity

Vero cells were exposed to increasing concentrations of monofunctional and Janus-type dendrimers functionalized with peptides, and the number of viable cells was determined using the 3-(4,5-dimethylthiazol-2-yl)-2,5-diphenyltetrazolium bromide (MTT) assay [72]. Vero cells were subcultured in 96-well plates at a seeding density of 2 × 10^4^ cells/well and treated with compounds 5.5 nM, 55 nM, 0.28 μM, 0.55 μM, 1.1 μM, 2.8 μM for 3 and 24 h. The medium was then gently aspirated, MTT solution (5 mg/mL) was added to each well, and cells were incubated for a further 3 h at 37 °C. The medium with MTT solution was removed, and the formazan crystals were dissolved with dimethyl sulfoxide. The absorption values were measured at λ_570_ using a Bio-Rad Microplate Reader (Bio-Rad Laboratories, Hercules, CA, USA). The viability of Vero cells in each well was reported respect to the control cells (untreated cells).

## Figures and Tables

**Table 1:** Peptide Sequences and dendrimer used.

Compounds	Peptide	Sequence	MW	Charge
**gH**	PrA-gH493-511	NH_2_-PrA-AAHLIDALYAEFLGGRVLT-CONH_2_	2124	−1
**Cys-gH**	Cys-gH493-511	Ac-C-AAHLIDALYAEFLGGRVLT-CONH_2_	2174	−1
**gB**	gB503-523-PrA	NH_2_- FARLQFTYNHIQRHVRDMEGR-PrA-CONH_2_	2769	+2
**DendrimerA**		Monofunctional dendrimer	3430	
**DendrimerB**		Bifunctional dendrimer	3043	

**Table 2:** ζ-Potential measurement of DendrimerB and DendrimerB + gB/gH.

	ζ-Potential (mV)	Std. Dev (mV)
**DendrimerB**	−11.0	±1.6
**DendrimerB + gB/gH**	5.17	±0.27
